# A combination of PARP and CHK1 inhibitors efficiently antagonizes MYCN-driven tumors

**DOI:** 10.1038/s41388-021-02003-0

**Published:** 2021-09-10

**Authors:** Stefano Di Giulio, Valeria Colicchia, Fabio Pastorino, Flaminia Pedretti, Francesca Fabretti, Vittoria Nicolis di Robilant, Valentina Ramponi, Giorgia Scafetta, Marta Moretti, Valerio Licursi, Francesca Belardinilli, Giovanna Peruzzi, Paola Infante, Bianca Maria Goffredo, Anna Coppa, Gianluca Canettieri, Armando Bartolazzi, Mirco Ponzoni, Giuseppe Giannini, Marialaura Petroni

**Affiliations:** 1 Department of Molecular Medicine, University La Sapienza, 00161 Rome, Italy; 2 Laboratory of Experimental Therapies in Oncology, IRCCS Istituto Giannina Gaslini, 16147 Genoa, Italy; 3 Pathology Research Laboratory, Sant’Andrea University Hospital, 00189 Rome, Italy; 4 Department of Experimental Medicine, University La Sapienza, 00161 Rome, Italy; 5 Department of Biology and Biotechnologies “Charles Darwin”, University La Sapienza, 00185 Rome, Italy; 6 Center for Life Nano- & Neuro-Science, Fondazione Istituto Italiano di Tecnologia (IIT), Rome, Italy; 7 Metabolic Pathology Lab, Ospedale Pediatrico Bambino Gesù, 00165 Roma, Italy; 8 Istituto Pasteur-Fondazione Cenci Bolognetti, 00161 Rome, Italy; 9 Present Address: Department of Biology, University Tor Vergata, 00173 Rome, Italy; 10 Present Address: Experimental Therapeutics Group, Vall d’Hebron Institute of Oncology (VHIO), Vall d’Hebron Research Institute (VHIR), Barcelona, Spain; 11 Present Address: Cellular Plasticity and Disease Group, Institute for Research in Biomedicine (IRB Barcelona), Barcelona Institute of Science and Technology (BIST), 08028 Barcelona, Spain

**Keywords:** Targeted therapies, Paediatric cancer, DNA damage checkpoints

## Abstract

MYCN drives aggressive behavior and refractoriness to chemotherapy, in several tumors. Since MYCN inactivation in clinical settings is not achievable, alternative vulnerabilities of MYCN-driven tumors need to be explored to identify more effective and less toxic therapies. We previously demonstrated that PARP inhibitors enhance MYCN-induced replication stress and promote mitotic catastrophe, counteracted by CHK1. Here, we showed that PARP and CHK1 inhibitors synergized to induce death in neuroblastoma cells and in primary cultures of SHH-dependent medulloblastoma, their combination being more effective in MYCN amplified and MYCN overexpressing cells compared to MYCN non-amplified cells. Although the MYCN amplified IMR-32 cell line carrying the p.Val2716Ala ATM mutation showed the highest sensitivity to the drug combination, this was not related to ATM status, as indicated by CRISPR/Cas9-based correction of the mutation. Suboptimal doses of the CHK1 inhibitor MK-8776 plus the PARP inhibitor olaparib led to a MYCN-dependent accumulation of DNA damage and cell death in vitro and significantly reduced the growth of four in vivo models of MYCN-driven tumors, without major toxicities. Our data highlight the combination of PARP and CHK1 inhibitors as a new potential chemo-free strategy to treat MYCN-driven tumors, which might be promptly translated into clinical trials.

## Introduction

MYCN deregulation is a recognized driver for numerous childhood and adulthood neuronal and nonneuronal tumors including neuroblastoma, medulloblastomas, rhabdomyosarcoma, neuro-endocrine prostate cancer, Wilms tumor, lymphoma and AML [1]. Mechanistically, it is often caused by genetic amplification, as seen in neuroblastoma and type 3 medulloblastoma. However, it may also depend on transcriptional upregulation, as in the case of Sonic Hedgehog (SHH)-dependent medulloblastoma [2]. MYCN deregulation is associated with aggressive behavior, which frequently leads to relapse even after multimodal chemotherapy [1]. MYCN amplification is an independent poor prognosis marker for many tumor types [1]. This is typically the case for MYCN amplified (MNA) neuroblastoma, accounting for about 50% of the high-risk cases sharing a 50% 5-year survival rate [3].The driving role of MYCN in cancer was confirmed in genetically engineered mouse models [4–8].

Although MYC inactivation is therapeutically effective in preclinical models [9], validated means to directly target MYCN in cancer patients are lagging behind. Thus, modulating pathways which MYCN-driven tumors are addicted to is an alternative strategy to cure patients affected by these tumors.

MYCN induces replication stress (RS) and a DNA damage response (DDR) [10–13] but also modulates the expression of a large set of DNA repair factors, which restrain the deleterious effects of replication-born DNA damage [12, 14–17]. Interfering with such RS-response (RS-R) and DDR proved effective in inducing cancer cell death, in MYCN-driven tumors.

Several compounds modulating the RS-R and DDR are being tested in preclinical cancer models and clinical trials [18–21]. Among them ATR, CHK1, and PARP inhibitors received increasing attention and are in advanced development. The ATR-CHK signaling pathway plays a central role in securing DNA replication and controlling RS via replication fork stabilization [22]. Its inhibition leads to destabilization of replication forks, which may collapse and generate DNA double strand breaks. Targeting the ATR-CHK1 pathway also impairs S and G2 checkpoints, as well as homologous recombination repair (HRR), causing an accumulation of excess DNA damage and cell death by replication or mitotic catastrophe, especially in cancer cells with intrinsically high RS or impaired DNA repair [22, 23]. CHK1 inhibitors are being tested in preclinical and clinical trials for the treatment of hematological and solid tumors, especially in combination with antimetabolites or other chemotherapeutics, although initial studies are reporting significant toxicities [24–26]. CHK1 inhibitors have also been tested in childhood cancer preclinical studies, in combination with chemotherapy or alone, with encouraging results [27–29]. A phase 1 clinical trial for CHK1 inhibition in pediatric cancer was recently completed [30].

Poly(ADP-ribose)polymerases (PARP) 1 and 2 participate in DNA repair via multiple pathways [31] and PARP inhibitors have been employed to enhance the activity of anticancer drugs and radiation in preclinical models [32]. The observation that BRCA1/2-defective cells are oversensitive to PARP inhibition [33, 34] led to the discovery that PARP inhibitors are synthetic lethal with an intrinsic dysfunction of the homologous recombination pathway. PARP inhibitors are now in clinical routine for BRCA-defective breast, ovarian and pancreatic cancer and are being tested in other tumors bearing defective DDR molecules, including ATM [19, 21]. Genomic alterations in DDR-associated genes frequently occur in high-risk neuroblastoma cases with chromosome 11q deletion, suggesting the potential utility of PARP inhibitors in these patients [35, 36] which is now being tested in a clinical trial [37].

We have recently shown that PARPs are overexpressed in MNA neuroblastoma, and that PARP inhibitors with high trapping properties enhance MYCN-dependent RS and activate S and G2/M checkpoints. Nonetheless, MYCN-driven cancer cells treated with PARP inhibitors enter mitosis with damaged DNA and undergo mitotic catastrophe, suggesting that increasing oncogene-dependent RS is another mechanism contributing to lethality by PARP inhibitors [38]. CHK1-dependent checkpoints protect MYCN-inducible cell models from mitotic catastrophe [38], which led us to speculate that PARP and CHK1 inhibitors might synergize in killing MYCN-driven tumors.

Here, we addressed this question by using multiple in vitro and in vivo preclinical models of MYCN-driven neuroblastoma and medulloblastoma.

## Results

### PARP inhibitors enhance the sensitivity to CHK1 inhibitors in MYCN-dependent tumor models

**Fig. 1:**
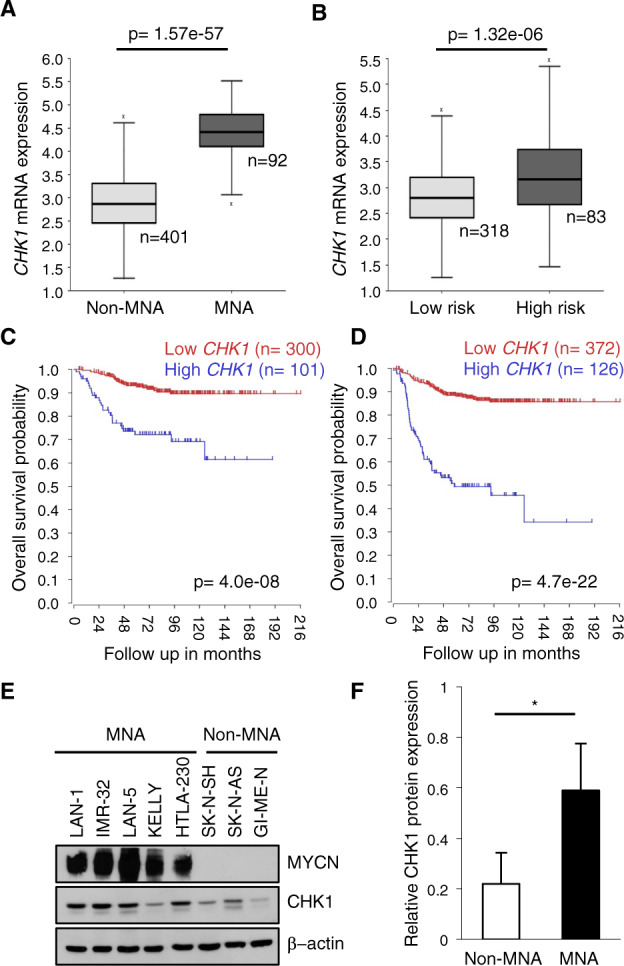
High CHK1 expression correlates with MYCN status and predicts clinical outcome in neuroblastoma patients. **A**–**D** Data analyses on the SEQC-498-RPM dataset performed on the R2-Genomics platform. Box plots of *CHK1* mRNA expression relative to MYCN status (**A**) and risk classification (**B**). Kaplan–Meier curves reporting overall survival with respect to *CHK1* mRNA expression in neuroblastoma patients (**C**) and in the non-MNA subset (**D**). The last quartile modus was used for cut-off determination with a minimum group size of 8 to determine the best *p* values. **E** Western blot (WB) analysis of whole cell lysates from the indicated non-MNA and MNA cell lines. Blots were probed with the indicated antibodies; β-actin was used as loading control. **F** Bar plot representing the mean CHK1 protein expression (+SD) in non-MNA and in MNA cells. Densitometry was performed using ImageJ software (ver. 1.51j8). *p < 0.05. *p* value was calculated by a two-sided Student’s *t* test.

*CHK1* is highly expressed in MNA compared to MYCN non-amplified (non-MNA) and in high-risk compared to low risk non-MNA neuroblastoma patients (Fig. 1A, B). High *CHK1* mRNA expression is associated with a reduced survival in neuroblastoma patients, independently from MNA (Fig. 1C, D), with the same trend we previously reported for *PARP1* and *PARP2* [38]. The higher CHK1 and PARP1 expression in MNA compared to non-MNA neuroblastoma is also recapitulated in the cell lines (Fig. 1E, F and our previous work [38]), which therefore represent good models to study the effects of combined PARP and CHK1 inhibition.

**Fig. 2:**
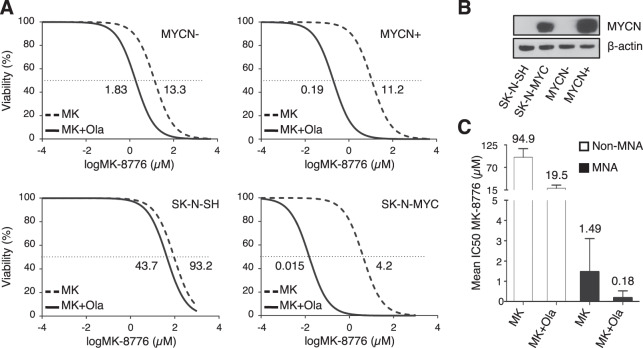
MK-8776/olaparib combination is more effective in MNA and MYCN-OE neuroblastoma models. **A** Viability assay performed on two syngeneic MYCN-OE cell models (inducible SHEP Tet21/N, MYCN± and SK-N-SH and SK-N-MYC pair) after continuous treatment with different doses of MK-8776 alone or in combination with olaparib (10 µM), for 48 h. Data are reported as mean (+SD) of three independent experiments. Cell viability was calculated as the percentage of untreated controls. MK-8776 IC50 values (µM) are reported close to each curve. **B** WB analysis performed on whole-cell lysates of MYCN-OE cells models for MYCN expression. β-actin was used as loading control. **C** Mean IC50s ( +SD) of MK-8776 dosed alone or in combination with olaparib in all non-MNA and MNA cells represented in supplementary fig. 1. IC50 mean values (µM) are reported above the bars.

The CHK1 inhibitor MK-8776 showed efficacy in inhibiting cell viability of MYCN-overexpressing (MYCN-OE) cells. Indeed, MK-8776 IC50 was lower in the stably transfected SK-N-MYC cells [39] compared to parental SK-N-SH cells and was reduced by MYCN in the inducible SHEP Tet21/N model (since now on MYCN + /MYCN− cells, Fig. 2A, B). Consistently, all MNA cells were more sensitive to CHK1 inhibition than non-MNA cells, with an average IC50 of 1.49 μM and 94.9 μM, respectively (Table 1, Fig. 2C and Fig. S1A). Most importantly, while the combination with a fixed dose (10 μM) of the PARP inhibitor olaparib sharply reduced MK-8776 IC50 in all cell lines we observed a more dramatic effect in MYCN-OE and MNA cells (Figs. 2 and S1), showing a 8.28-fold versus a 4.87-fold reduction MK-8776 IC50 in MNA cells compared to non-MNA cells (Fig. 2C). In combination with olaparib the MK-8776 IC50 systematically fell below the micromolar range (0.005–0.8 μM) in MNA cell lines, being IMR-32 and LAN-1 the most and the less sensitive MNA cells, respectively (Table 1). Most importantly, in all cell lines we demonstrated relevant synergy scores between MK-8776 and olaparib, although they were much higher in MNA compared to non-MNA cells (Fig. S2A). Treatment of IMR-32 cells with differing drug combinations including talazoparib, another PARP inhibitor [40], or PF-00477736, a different CHK1 inhibitor provided similar outcome in viability assays (Fig. S1B).

**Table 1:** IC_50_ (μM concentrations) of MK-8776 (MK) and MK-8776+olaparib (MK + Ola) in NB cell lines.

Cell line	MYCN status	MK	MK + Ola
LAN-1	Amp.	3.01	0.80
IMR-32	Amp.	3.58	0.005
LAN-5	Amp.	0.11	0.01
KELLY	Amp.	0.42	0.04
HTLA-230	Amp.	0.31	0.06
SK-N-AS	Non amp.	118.9	10.9
SK-N-SH	Non amp.	85.0	22.2
GI-ME-N	Non amp.	80.8	25.4

Overall, these data indicate that CHK1i and PARPi synergize to efficiently inhibit cell growth and/or survival of MYCN-OE and MNA cell lines.

### The CHK1i/PARPi combination induces DNA damage and cell death in MYCN-dependent tumor models

**Fig. 3:**
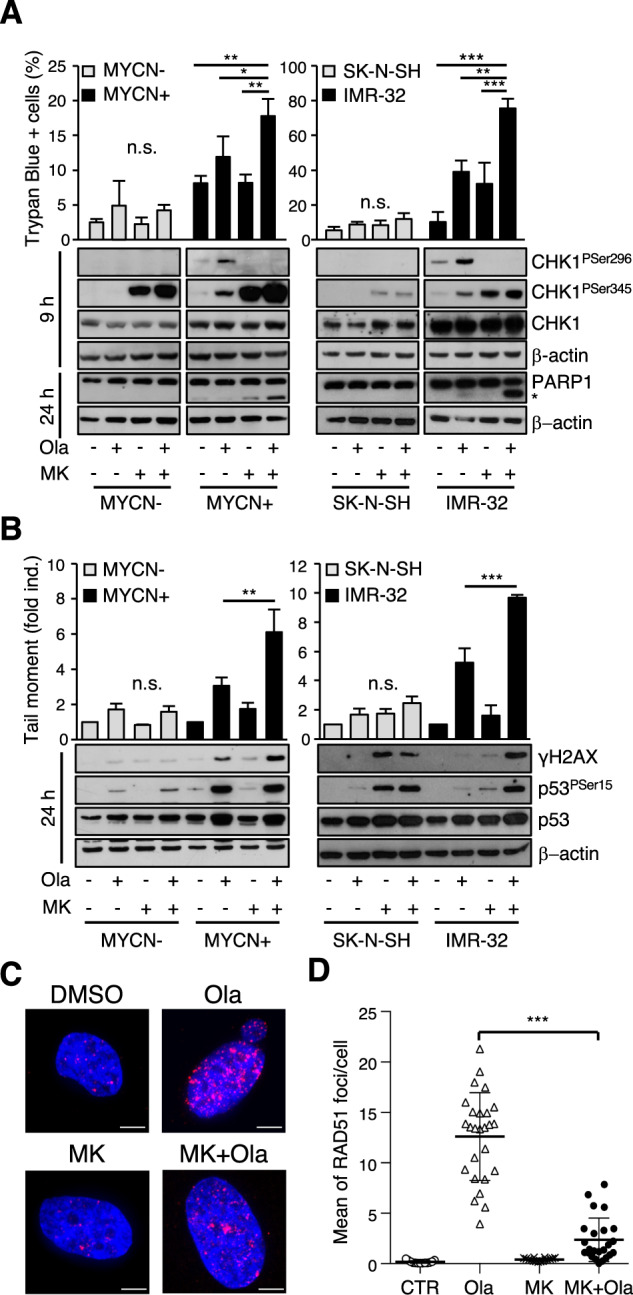
MK-8776/olaparib combination induces DNA damage and cell death in MYCN-OE and MNA cells. **A** Upper panels, cell death monitored by trypan-blue exclusion assay in the indicated cells treated with olaparib, MK-8776 and their combination, for 48 h. Data are reported as mean (+SD) of three independent experiments. **A** Lower panels, WB analysis of whole cell lysates from the indicated cells performed after 9 and 24 h of treatment. Blots were probed with the indicated antibodies; β-actin was used as loading control. Data are representative of at least three replicates. The asterisk indicates the 89-kDa PARP1 cleaved fragment. **B** Upper panels, Neutral comet assay performed on the indicated cells treated with olaparib, MK-8776 and their combination, for 24 h. DNA damage is quantified by tail moment. Data are reported as mean (+SD) of three independent experiments. **B** Lower panels, whole cell lysates were analyzed by WB for the expression and/or phosphorylation of the indicated proteins; β-actin was used as loading control. Data are representative of at least three replicates. **C** Representative Z-stack images captured by confocal microscopy of RAD51 foci in MYCN + cells treated with olaparib, MK-8776 and their combination, for 24 h. Scale bar: 5 µm. **D** Dot plot representing the mean of RAD51 foci/cell ± SD in MYCN + cells treated as above. Each dot represents a field of at least 50 cells. The graph is representative of two independent experiments. *p* values were calculated by ANOVA (*p < 0.05, **p < 0.01, ***p < 0.001).

Because the maximum inhibitory effect was reached with a combination of a suboptimal dose (1 μM) of MK-8776 and 10 μM olaparib in all cell lines (Fig. S2), we used these concentrations for further in vitro experiments. Under these conditions, MK-8776 modestly affected cell death, while olaparib displayed some effects on MYCN-OE and MNA cells (Figs. 3A and S2), as previously reported [38]. Most importantly, the MK-8776/olaparib combination significantly increased the levels of cell death in MYCN-OE and MNA cells, but not in non-MNA cells, as revealed by the trypan-blue exclusion assay and by detection of the PARP cleaved fragment (Fig. 3A and Fig. S3). Mechanistically, olaparib activated the S-phase checkpoint in MYCN + cells and induced Ser296 and Ser345 CHK1 phosphorylation in MNA and MYCN-OE cells lines, revealing an increase in RS (Fig. S4 and Fig. 3A). In combination treatments, MK-8776 inhibited CHK1 activity as assessed by Ser296 phosphorylation (Fig. 3A and S3) and both PF-00477736 or MK-8776 prevented the execution of the S-phase checkpoint induced by olaparib, favoring cell cycle progression, in MYCN + cells (Fig. S4). As expected, olaparib induced significant accumulation of DNA double strand breaks and Rad51 foci in MYCN-OE and MNA cells (Fig. 3B, C, D). MK-8776 suboptimal dose did not significantly affect the level of DNA damage or cell death as a single agent, but almost doubled the effects of olaparib in the combined treatment, while also preventing Rad51 foci formation, in MYCN-OE and MNA cells (Fig. 3B, C, D) suggesting a CHK1i-dependent impairment of HRR.

Of interest, MK-8776 alone and the combined treatment induced γH2AX and phosphorylated p53 at Ser15 in SK-N-SH, but not comet assay-detectable DNA double strand breaks, possibly due to the accumulation of replication intermediates or other non-lethal types of DNA damage (Fig. 3B, right panel).

Overall, these results indicate that the synergic killing effect induced by the combination of PARPi and CHK1i is most likely due to a PARPi-dependent increase in RS coupled with the inhibition of the CHK1-driven HRR and checkpoints, resulting in inappropriate cell cycle progression with unrepaired DNA damage and cell death.

### IMR-32 sensitivity to MK-8776/olaparib combination does not depend on their ATM mutant status

**Fig. 4:**
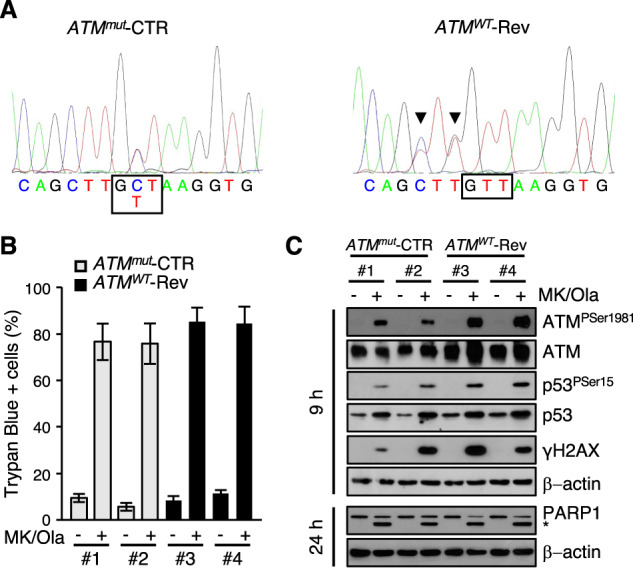
The effect of MK-8776/olaparib combination on IMR-32 cells does not depend on p.Val2716Ala ATM mutation. **A** Electropherograms representing DNA sequences obtained from control (*ATM*^*mu*t^-CTR) and CRISPR/Cas9 reverted (*ATM*^*WT*^-Rev) IMR-32 clones. Black boxes indicate the mutated/reverted codon; arrowheads indicate blocking mutations in the reverted clones. **B** Cell death monitored by the trypan-blue exclusion assay on *ATM*^*mut*^-CTR and *ATM*^*WT*^-Rev clones treated with vehicle or MK-8776/olaparib (MK/Ola) combination, for 48 h. Data are reported as mean (+SD) of three independent experiments. **C** WB analysis of whole cell lysates obtained from the indicated cells after 9 and 24 h of treatment. Blots were probed with the indicated antibodies and β-actin was used as loading control. Data are representative of at least three replicates. The asterisk indicates the 89 kDa PARP1 cleaved fragment.

Due to the loss of multiple genes involved in HRR, including ATM, PARP inhibitors have been proposed as therapeutic agents for neuroblastoma patients with 11q deletion [35, 36]. A putatively pathogenic ATM mutation (the c.8147 T > C; p.Val2716Ala) was described in the IMR-32 cell line [41–43]. To address whether it could be responsible for the high sensitivity of these cells to the MK-8776/olaparib combination, we reverted it to WT state via the CRISPR/Cas9 technology (Fig. 4A). Two independent *ATM*^*wt*^ reverted clones showed a slightly increased ATM expression compared to *ATM*^*mut*^ controls (Fig. 4C). They also showed a slightly increased ATM, p53 and H2AX phosphorylation in response to MK-8776/olaparib combination, but no decrease in cell death (Fig. 4B, C) suggesting that the p.Val2716Ala ATM mutation is not responsible for the high sensitivity of IMR-32 cells to the combination treatment.

### MK-8776/olaparib combination reduces tumor growth in preclinical neuroblastoma mouse models

**Fig. 5:**
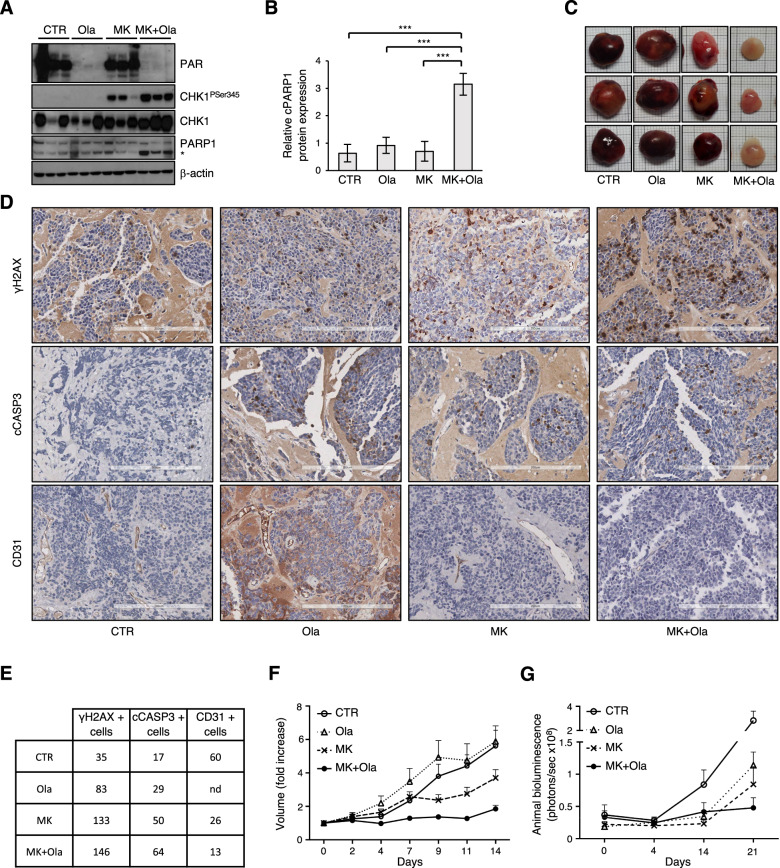
MK-8776/olaparib combination reduces tumor growth in subcutaneous and orthotopic mouse models of neuroblastoma. **A** WB analysis of tumor lysates explanted from IMR-32 xenografts intraperitoneally injected with vehicle (CTR), olaparib (Ola), MK-8776 (MK) and their combination (MK + Ola) for 30 h (*n* = 3/group). Blots were probed with the indicated antibodies and β-actin was used as loading control. The asterisk indicates the 89 kDa PARP1 cleaved fragment. **B** Bar plot representing the ratio between cleaved versus full length PARP1 expression assessed by densitometry (means ± SD) of the WB in (5**A**). **C** Tumors explanted from IMR-32 xenografts after 2 weeks (*n* = 3/group). **D** Representative images of IHC analysis with the indicated antibodies in tumor samples explanted after 5 days of treatment; magnification (×132). Scale bar: 200 µm. **E** Mean counts of γH2AX, cleaved caspase 3 and CD31 positive cells detected on four high power fields (HPF) for each of four biological replicates/group. nd not determined. **F** Evaluation of tumor growth in IMR-32 xenograft treated as above for 2 weeks; data were expressed as mean volume + SEM (*n* = 7/group, Ola *n* = 6). **G** Evaluation of tumor growth in nude mice orthotopically inoculated with IMR-32-luc cells and treated as above for three weeks. Tumor growth was monitored by bio-luminescence imaging (BLI) at the indicated times. Plot representing the photon counts in the tumor region of interest (ROI). Data were reported as mean + SEM (*n* = 10/group). For all experiments *p* values were calculated by ANOVA (*p < 0.05, **p < 0.01, ***p < 0.001).

Next we tested the effects of suboptimal doses of MK-8776 (compared to those previously used in neuroblastoma xenografts [29]) in combination with olaparib on IMR-32 subcutaneous xenografts. In a pilot experiment, six animals for each group were sacrificed after 30 h (*n* = 3) or 14 days (*n* = 3) of treatment. Analysis of parylation and Ser345 CHK1 phosphorylation indicated that all drugs reached the tumors and were effective (Fig. 5A). MK-8776/olaparib combination significantly increased PARP cleavage (Fig. 5A, B) and impaired tumor growth (Fig. 5C), compared to controls and single agents. Moreover, tumors treated with the combination of drugs appeared far less bloody and hemorrhagic (Fig. 5C), suggesting a reduced vascularization. In a second experiment, four animals/group were sacrificed after 5 days of treatment and 7 were maintained for survival analysis. While we did not observe any significant reduction in proliferation-index as assessed by Ki67 immunostaining in any treated sample (not shown), all drug-treated tumors showed an increase of γH2AX and cleaved caspase-3 positive cells compared to vehicle-treatement (Fig. 5D, E). Moreover, the number of CD31 positive cells was reduced by MK-8776 compared to controls (Fig. 5D, E), while it was not assessable in olaparib-treated tumors due to a recurrent aspecific interstitial background (Fig. 5D). Importantly, the highest number of γH2AX and cleaved caspase-3 and the lowest number of CD31 positive cells was invariably found in mice treated with the combined therapeutic approach (Fig. 5D, E). Consistently, MK-8776/olaparib-treated tumors grew at a significantly lower rate compared to control or single agent-treated tumors; MK-8776 alone also had a better outcome compared to the control (Fig. 5F, and S5A). At later time points, all tumors increased in size. Nonetheless, the MK-8776/olaparib combination prolonged the median survival (39 days) compared to both vehicle (30 days) and single agents (32 days) (Fig. S5B).

**Fig. 6:**
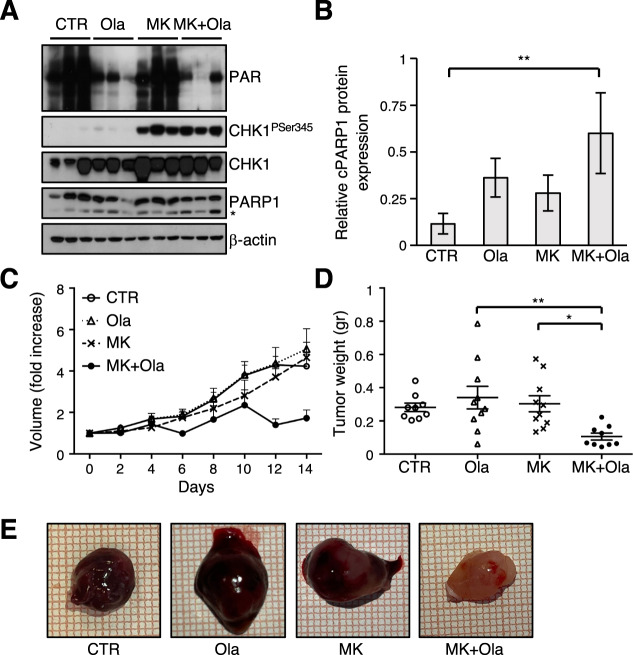
MK-8776/olaparib combination reduces tumor growth in LAN-5 subcutaneous xenograft model. **A** WB analysis of tumor lysates explanted from LAN-5 xenografts treated for 30 h (*n* = 3/group). Blots were probed with the indicated antibodies and β-actin was used as loading control. The asterisk indicates the 89 kDa PARP1 cleaved fragment. **B** Bar plot representing the ratio between cleaved versus full length PARP1 expression assessed by densitometry (means ± SD) of the WB in (6**A**). **C**–**D** Evaluation of tumor growth and weight in LAN-5 xenografts after 2 weeks of treatment; data were expressed as mean volume + SEM and mean weight ± SEM (*n* = 10/group, CTR and MK + Ola *n* = 9). **E** Representative images of tumors explanted after 2 weeks of treatment.

Notably, similar data were also obtained in a LAN-5 subcutaneous xenograft model (Fig. 6).

To test the effects of the combined treatment at an orthotopic site, we implanted IMR32-luc cells in the mice’s adrenal glands. After 2 weeks of treatments (day 14), we observed a reduction in bioluminescence in tumor-bearing mice treated with olaparib or MK-8776, administered alone and in combination versus vehicle-treated controls (Fig. 5G). After 3 weeks of treatment (day 21), tumor reduction became statistically significant, compared to controls, for both single agents and their combination, which led to the greatest and most significant reduction (Fig. 5G and S5C). Moreover, MK-8776/olaparib combination led to a prolonged survival, with a significant increase versus both vehicle (*P* = 0.0192) and single agent olaparib (*P* = 0.0275) (Fig. S5D).

In a similar experiment, no weight loss and no major tissue damage in liver, spleen, heart and kidney was evidenced in all treatment groups as well as no chronic toxicities emerged, as indicated by the quantification of the clinical chemistry and hematological markers (Fig. S6). Thus, combined inhibition of PARP and CHK1 has a potent effect in limiting tumor growth in subcutaneous and orthotopic MNA neuroblastoma models, without major toxicities.

### MK-8776/olaparib combination inhibits tumor growth in a SHH-dependent medulloblastoma model

**Fig. 7:**
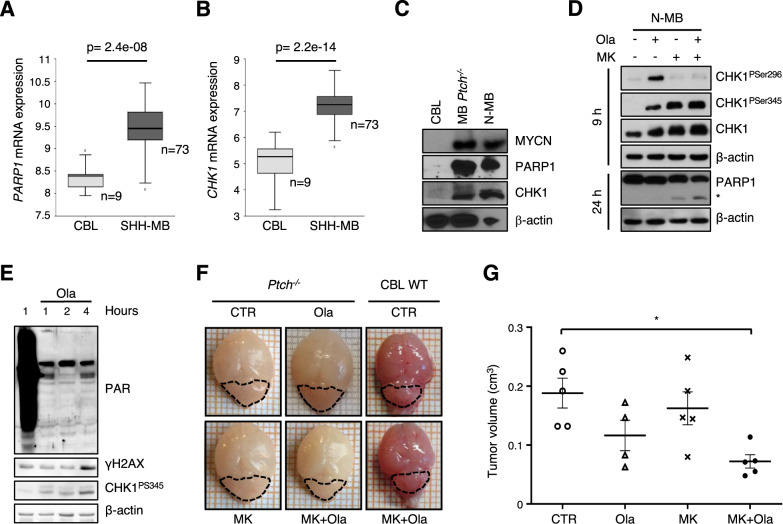
MK-8776/olaparib combination impairs tumor growth in in vitro and in vivo SHH-dependent medulloblastoma models. **A**–**B** Data analyses on medulloblastoma and normal cerebella datasets performed on the R2-Genomics platform. Box plots of *PARP1* (**A**) and *CHK1* (**B**) expression relative to normal cerebellum (CBL) and SHH-type medulloblastoma patients (SHH-MB). **C** WB analysis performed on whole extracts of young adult (P21) WT mice cerebella (CBL), Ptch-KO mice cerebella (MB *Ptch*^*−/−*^) and on extracts from neurosphere cultures obtained from a Ptch-KO medulloblastoma (N-MB). **D** WB analysis of whole-cell lysates of N-MB neurospheres treated for 9 and 24 h. Blots were probed with the indicated antibodies; β-actin was used as loading control. The asterisk indicates the 89-kDa PARP1 cleaved fragment. Data are representative of three replicates. **E** WB analysis of cerebella explanted from P5 *Ptch*^*−/−*^ mice after i.p. injection of vehicle or olaparib. Blots were probed with the indicated antibodies; β-actin was used as loading control. **F** Representative images of the cerebella explanted at P9 from *Ptch*^*−/−*^ or WT mice, treated daily for 4 days. Dotted lines highlight the size/shape of the cerebella. **G** Dot plots representing the mean tumor volume (±SEM) of mice treated as above (*n* = 5/group, Ola *n* = 4). *p* values were calculated by ANOVA (*p < 0.05).

SHH-dependent medulloblastomas (SHH-MBs) are associated with, and dependent on, an increased expression of MYCN [2]. Interestingly, we observed higher *PARP1* and *CHK1* expression in SHH-MB tissues compared to normal cerebellum in humans and in *Ptch*^*−/−*^ mice as well as in a *Ptch*^*−/−*^ medulloblastoma primary cultures (N-MB) (Fig. 7A, B, C). In these cells, MK-8776/olaparib combination induced cell death more efficiently compared to single treatments, as indicated by the morphology of the neurospheres (not shown) and by PARP1 cleavage (Fig. 7D).

Next, we tested the MK-8776/olaparib combination on the growth of SHH-MBs spontaneously arising in hGFAP-cre mediated *Ptch-KO (Ptch*^*−/−*^*)* mice [44]. These mice exhibited a dramatic expansion of both the rhombic lip and the EGL already at E16.5 and, after birth, when WT cerebellum was characterized by few rows of GNPs on its surface, the *Ptch*^*−/−*^ cerebellum was encompassed by a thick, disorganized EGL [44]. Olaparib inhibited parylation and induced CHK1 phosphorylation already 1 h after intraperitoneal (ip) injection, while accumulation of γH2AX was clearly detectable after 4 h, in P5 *Ptch*^*−/−*^ cerebellar extracts (Fig. 7E), indicating that olaparib can cross the blood-brain barrier to reach medulloblastoma tissue. Therefore, starting from P5, we treated *Ptch*^*−/−*^ mice daily with vehicle, olaparib, a suboptimal dose of MK-8776 or their combination, for 4 days. At P9, mice were sacrificed to measure the size of the cerebellum. MK-8776/olaparib combination significantly reduced the enlargement of the cerebellum compared to control or treatment with single agents, while it did not affect the volume of the developing cerebellum in WT mice (Fig. 7F, G). Accordingly, we observed a remarkable reduction in tumor cell density in the cerebella of MK-8776/olaparib- compared to control- or single agents-treated mice (Fig. S7).

## Discussion

MYCN-dependent tumors mostly occur as aggressive and deadly neoplasia, very poorly responsive to current therapies. Since clinically validated strategies to directly target MYCN are not yet available, the identification of alternative vulnerabilities of these tumors is urgently needed. In this light, targeting MYCN-dependent RS-R is emerging as a potentially useful strategy [10, 12, 14, 15, 38]. Here, we report that a chemo-free approach combining CHK1 and PARP inhibitors antagonizes the growth of MYCN-driven tumors in vitro and preclinical models in vivo, without major toxicities. Considering the advanced state of development and testing of the drugs, this strategy could be promptly translated into clinical trials.

Due to its involvement in RS-R and DDR, inactivation of the ATR-CHK1 signaling pathway leads to accumulation of excess DNA lesions and death due to S-phase or mitotic catastrophe, especially in cancer cells with high RS or impaired DNA repair [20]. In example, MYC oncogenic power was reported to be strictly addicted to the ATR-CHK1 pathway [45]. MYCN-driven tumors should also be addicted to such pathway. Indeed, the effectiveness of CHK1 inhibitors in preclinical neuroblastoma and medulloblastoma models and their enhanced activity in MYCN-driven tumors has been reported [45]. Here, we highlighted a higher CHK1 expression in MNA compared to non-MNA human neuroblastoma, and in SHH-type medulloblastomas compared to normal cerebella, consistent with an increased efficacy of CHK1i in MNA and in MYCN-OE. Of utmost importance, even suboptimal doses of CHK1i in combination with olaparib could still efficiently impair PARPi-induced and CHK1-dependent S-phase checkpoint and HRR, leading to the accumulation of RS-born DNA damage, DDR and cell death in MNA and MYCN-OE cells, providing a mechanistic explanation to the effects of the drug combination and suggesting potential clinical benefit by PARPi/CHK1 cotreatment. Most importantly, suboptimal doses of MK-8776 plus olaparib most efficiently antagonized the growth of different MNA neuroblastoma preclinical models in vivo, including subcutaneous and orthotopic xenografts. MK-8776/olaparib-treated tumors revealed a high rate of apoptosis and reduced tumor vascularization. This effect was also noted in MK-8776 single treatment and was previously observed in neuroblastoma xenografts treated with the CHK1 inhibitor prexasertib [28], suggesting that multiple mechanisms may contribute to tumor growth inhibition. The combination of low doses of MK-8776 plus olaparib efficiently inhibited also the growth of SHH-driven medulloblastoma in vitro and in vivo, confirming its efficacy in a different MYCN-driven tumor model. This experiment also indicated that olaparib can reach intracranial tumor tissue, as previously demonstrated for MK-8776 [46], and that the combined treatment does not affect postnatal cerebellar development.

Of relevance, with MK-8776/olaparib cotreatment we did not detect significant organ-specific or systemic toxicities, which are often seen in pharmacological associations of CHK1 inhibitors with DNA damaging drugs. CHK1 inhibitors alone or in combination with chemotherapy are being tested in phase1/2 clinical trials in adult and pediatric solid and hematological tumors, but toxicity issues emerged, which might limit their development and employment [24–26, 30]. Our data clearly indicates that pharmacological combinations with PARP inhibitors might improve the therapeutic index of CHK1 inhibitors by reducing dose-related side effects.

The synergic effects of PARP inhibitors with drug targeting of the ATR-CHK1 pathway on pediatric tumors was also observed by Southgate et al., who reported on the activity of the ATR inhibitor VE-821 and olaparib on four neuroblastoma cell lines [47], although a MYCN dependence was not clearly addressed in this study. Comparative studies in vivo will be required to address which of the two combination may provide best advantages together with lower side effects.

Results of a phase 1 trial of olaparib in pediatric patients with refractory solid tumors [37] is shortly expected. The trial was supported by the observation that 11q deletion affecting multiple genes relevant for homologous recombination (such as ATM) and/or their point mutations, were reported to putatively confer synthetic lethality with PARP inhibitors [35, 36]. Interestingly, IMR-32 cells, which we found most sensitive to MK-8776/olaparib combination, carry the putatively pathogenic p.Val2716Ala ATM mutation [41–43]. However, this is not responsible for their high sensitivity to PARPi/CHK1i combination, as demonstrated by CRISPR/Cas9-based genetic correction of the ATM mutation. This is consistent with MNA and MYCN-OE cells being oversensitive to PARP inhibitors due to the consequence of an enhanced RS and accumulation of replication-born DNA damage, whose consequences are further exacerbated by inhibition of cell cycle checkpoints via CHK1i.

Emerging evidence indicates that enhanced RS plays a central role in the activation of the immune response and could serve as a predictive marker for immunotherapy [20]. The effects of PARP inhibitors have been linked to modulation of the immune response, suggesting their use in combination with immune checkpoints inhibitors [48]. It is possible that additional benefits could be obtained by a combined PARP and CHK1 inhibition in immunocompetent mouse models. This hypothesis, not investigated here, deserves further attention in future work.

In conclusion, our findings indicate that olaparib enhances the therapeutic index of CHK1 inhibitors in MYCN-driven tumors and suggests that clinical trials should be undertaken to address the safety and efficacy of this chemo-free association. Since chromosome 11q deletion associated DDR-defects are anti-correlated with MNA in aggressive neuroblastoma cases, these data suggest that PARP inhibitors might find successful applications in both tumor subtypes.

## Materials and methods

### Public dataset gene expression analysis

R2-Genomics analysis and visualization platform (http://r2.amc.nl) was used to investigate molecular and clinical features of neuroblastoma and medulloblastoma patients relative to *CHK1* and/or *PARP1* expression. Neuroblastoma dataset: SEQC-498-RPM, GSE62564; medulloblastoma dataset: (SHH)-Pfister −73-MAS5.0, GSE49243; normal cerebellum dataset: Roth-9-MAS5.0, GSE3526. Data were downloaded from the website and formatted for publication.

### Cell lines and culture conditions

The stable cell lines used in this work were acquired by different sources and cultured with standard procedures. All cell lines were validated by short tandem repeat (STR) DNA analysis (LGC Standards, Teddington, Middlesex, UK). MYCN-inducible SHEP Tet21/N cells were cultured with doxycycline (2 μg/mL) (MYCN-) or without doxycycline (MYCN + ) and validated for MYCN induction [49]. Medulloblastoma neurospheres culture (N-MB), were generated following a protocol described in [50]. For more details, please refer to supplemental information.

### Drug treatments for viability assays, IC50s and synergy determination

Cells were seeded in 96-well plates at low density and treated (48 or 72 h) with the following drugs: CHK1 inhibitors MK-8776 (SCH 900776, Selleckchem, Houston, TX, USA) or PF-00477736 (Sigma Aldrich) at different doses (0.01, 0.1, 1, 10 μM); PARP inhibitors olaparib (AZD2281, Selleckchem, 1, 10 μM) or talazoparib (BMN-673, MedChem express, Monmouth Junction, NJ, USA, 10 μM). Viability assay was performed using the RealTime-Glo MT Cell Viability Assay kit (#G9711, Promega Corporation, Madison, WI, USA), using a Glomax Multidetection Luminometer, according to manufacturer’s protocol. Luminescence data were normalized on controls and plotted as the percentage of viability over the logarithmic concentration of CHK1 or PARP inhibitor. IC50 values were calculated using the GraphPad Prism 7 software (GraphPad, La Jolla, CA). Dose-response matrices and the synergistic score between compounds were obtained using the SynergyFinder tool. The delta scores were calculated as indicated (https://www.synergyfinder.fimm.fi/).

### Cell proliferation, cell death analysis, cell cycle FACS analysis and comet assay

Subconfluent cells were treated with MK-8776 (1 μM) and/or olaparib (10 μM) for 24 or 48 h, unless otherwise specified. Alive and dead cells were counted by the trypan-blue exclusion assay. At least 200 cells/sample were counted in triplicate experiments. For FACS analysis, cells were treated with olaparib, PF-00477736 (1 μM), MK-8776 or their combinations for 9 h. The DNA of harvested cells was stained with 7-AAD (10 μg/ml), as reported [38] and analyzed using a BD LSRFortessa (BD Biosciences, San Jose, CA, USA). The percentages of cells in different phases of the cell cycle were determined using the FlowJo V9.3.2 computer software (TreeStar, Ashland, OR, USA). At least 20,000 events for each sample were acquired. Neutral comet assay was conducted as described [16].

### Immunofluorescence

Cells were permeabilized in 0.5% Triton X-100 on ice for 8 min, fixed in 3% PFA − 2% sucrose in phosphate-buffered saline (PBS) for 10 min at RT, blocked in 3% BSA in PBS for 15 min at RT, incubated with anti-RAD51 antibody (ab133534, Abcam, Cambridge, UK) for 1 h at 37 °C and revealed with AlexaFluor-594 secondary antibody (Life Technologies). Images were acquired on a LEICA DM 2500 microscope or alternatively on a XLight V3 spinningdisk confocal unit (CrestOptics s.p.a.) coupled to an IX73 inverted microscope (Olympus). Microscopy data were collected at the CLN2S@Sapienza Imaging Facility of the Istituto Italiano di Tecnologia. RAD51 foci number was counted by a self-made CellProfiler pipeline and data were analyzed by GraphPad Prism 9 (GraphPad, La Jolla, CA) [51].

### Protein extraction and western blot

Total protein extracts were obtained and separated as described [52–54]. Primary antibodies and peroxidase-conjugated secondary antibodies are listed in the supplemental information. Immunoreactive bands were visualized by enhanced chemoluminescence (Advansta Inc., Menlo Park, CA, USA).

### CRISPR/Cas9

sgRNA specific to the c.8147 T > C mutation (mutATM-sgRNA) and the single-stranded donor oligonucleotides (ssODN) were designed using the informatics platform Benchling (Benchling, 2019, https://benchling.com). PAM sequence (AGG) was located three nucleotides downstream of the c.8147 T > C mutation. ssODN contained the reverted form of the c.8147 T > C mutation and two additional silent “blocking mutations” on the seed sequence to avoid Cas9 re-cutting [55]. The mutATM-sgRNA and scRNA (Origene, Rockville, MD, USA) were cloned into the LentiCRISPR v2 plasmid (Addgene plasmid #52961). IMR-32 cells were co-transfected with mutATM-sgRNA or scRNA-LentiCRISPR v2 plasmid and ssODN using Lipofectamine2000 Transfection Reagent (#11668027 Thermo Fisher Scientific, Waltham, MA, USA) according to the manufacturer’s instructions. The selection and validation procedure for the ssODN positive clones is described in Supplemental Information.

### In vivo studies with MK-8776/olaparib combination

For in vivo experiments, olaparib and MK-8776 dissolved in DMSO and diluted into 2-hydroxy-propyl-β-cyclodextrin (2-HPβC)/PBS solution were administered by ip injection at 50 mg/Kg and 25 mg/kg, respectively.

For the subcutaneous xenograft, IMR-32 cells (4 × 10^6^ cells) and LAN-5 (2.5 × 10^6^ cells) were injected subcutaneously in the posterior flanks of BALB/c nude mice (Nu/Nu, Charles River Laboratories, Lecco, Italy). Before treatment animals were randomly divided into four groups and injected with vehicle, olaparib, MK-8776 or their combination and different sets were used to test the early biochemical responses or the long-term efficacy of the drugs. In all cases, tumor growth was monitored by caliper size measurement and calculated by the formula length × width × 0.5 × (length + width).

In survival studies, mice were sacrificed when the tumor size reached 2 cm^3^ or, at latest, on day 40. The time span between the first treatment and the sacrifice was used as a surrogate for survival. MS (50% survival) was determined by GraphPad Prism 7 software (La Jolla, CA). %ILS was calculated by the formula: (median survival of treated mice − median survival of control mice)/median survival of control mice.

For orthotopic xenografts, 5-weeks-old female athymic Foxn1nu mice (Envigo, Bresso, Italy) were subjected to laparotomy and IMR-32-luc cells (with 1 × 10^6^) were transplanted into the left adrenal gland capsule, as previously described [56]. Luciferase activity was visualized by in vivo bio-luminescent imaging (BLI, IVIS Caliper Life Sciences, Hopkinton, MA) after a 10 min incubation with 150 µg/mL of D-luciferin (Caliper Life Sciences), as described [57]. Tumor-bearing mice were randomized into four groups for efficacy studies and systemic toxicity evaluation. For the systemic toxicity studies, blood samples were collected 48 h after the last treatment for clinical chemistry and hematological evaluations. Finally, mice were sacrificed and tumors and healthy organs (liver, spleen, heart, kidney) were collected for histopathological examination.

For the medulloblastoma mouse model, post-natal day 5 (P5) human GFAP promoter-driven CRE-mediated *Ptch-KO (Ptch*^*−/−*^*)* mice [44] were ip injected with drugs/drug combination. At P9, animals were sacrificed and cerebella were measured by caliper and collected for histopathological examination.

Animal experiments were approved by the ethical committee of the Italian Ministry of Health (protocol n.: n°379/2016-PR and n.: 661/2016-PR) in compliance with the “ARRIVE” guidelines (Animals Research: Reporting in Vivo Experiments). More details are in supplemental information.

### Immunohistochemistry

IHC was performed according to the manufacturer’s instructions with the following primary antibodies: a rabbit monoclonal antibody to phosphorylated-H2A.XSer139 #9718 (20E3, Cell Signaling Technology, Massachusetts, USA), a rat monoclonal antibody to CD31 #DIA310 (PECAM-1, clone SZ31, Dianova GmbH, Hamburg, Germany), a rabbit polyclonal antiserum to cleaved caspase-3 #9661 (Asp 175, Cell Signaling Technology, Massachusetts, USA), a mouse monoclonal antibody to KI-67 #M7240 (Clone MIB-1, Agilent, California, USA).

Immunohistochemical staining was assessed and scored by two independent pathologists who were blinded to the clinicopathological data. Discrepancies were resolved by consensus. More details are in supplemental information.

### Statistical analysis

For in vitro experiments data are expressed as mean ± standard deviation (SD). The *p* value was determined using one-way analysis of variance (ANOVA) with Tukey’s Multiple Comparison Test or two-sided Student’s *t* test by the GraphPad Prism 7 software (La Jolla, CA). For in vivo studies data are expressed as mean ± standard error (SEM). In order to evaluate differences between treatments we performed one-way ANOVA with Tukey’s Multiple Comparison Test; survival curves were drawn as Kaplan–Meier Cumulative Proportion Surviving graphs, and corresponding *p* values were calculated by the use of the log-rank (Mantel–Cox) test. Prism 7 software was also used to calculate the median survival. The sample size determination was accounted on the need for statistical power.

## Supplementary information

Supplemental information

Supplementary figures
