# Supplementary material for: A combination of PARP and CHK1 inhibitors efficiently antagonizes MYCN-driven tumors
Source: Oncogene. 2021 Sep 10;40(43):6143–52. doi: 10.1038/s41388-021-02003-0 (PMC8553625; doi:10.1038/s41388-021-02003-0)
Supplement: Supplementary file 1 — Supplemental information [file 41388_2021_2003_MOESM1_ESM.pdf]

SUPPLEMENTAL INFORMATIONS

Cell lines and culture conditions

SK-N-AS, SK-N-SH and SK-N-MYC cells were a gift of Dr. Carol J. Thiele (NCI, Bethesda, MD); GI-ME-N were acquired from Banca Biologica and Cell Factory (Genoa, Italy; www.iclc.it). IMR-32 were acquired from European Collection of Cell Cultures (Porton Down, UK; www.ecacc.org.uk); LAN-1 were a kind gift of Dr. Nicole Gross (Department of Pediatrics, University Hospital, Lausanne, Switzerland); LAN-5 and Kelly were acquired from Deutsche Sammlung von Mikroorganismen und Zellkulturen (Braunschweig, Germany; www.dsmz.de); HTLA-230 were from the IRCCS Istituto G. Gaslini, Genoa, Italy. SHEP Tet21/N cell line, received from Dr. Schwab, DKFZ, Heidelberg, Germany, is a non-MNA neuroblastoma cell line in which exogenous MYCN expression is under the control of a tetracycline responsive promoter (Tet-off system). SK-N-MYC is a non-MNA neuroblastoma cell line derived from SK-N-SH in which MYCN is stably transfected by pREPcNMYC2 plasmid (Judware R, Culp LA. *Oncogene* 1995; 11: 2599-2607). SK-N-AS, GI-ME-N, LAN-5, Kelly and SHEP Tet21/N cells were cultured in RPMI 1640 (#R0883 Sigma Aldrich, St. Louis, MO, USA), supplemented with 10% FBS (#10270-106 GIBCO), 1% penicillin-streptomycin (#P4333 Sigma Aldrich) and 2 mM L-glutamine (#G7513 Sigma Aldrich). For SHEP Tet21/N cells, G418 (200 µg/ml, #4941803 SERVA) and hygromycinB (90 µg/ml, #H7772 Sigma Aldrich) were added to the medium. LAN-1 were cultured in the same medium supplemented with 1% non-essential Amino Acid (#M7145 Sigma Aldrich). SK-N-SH and SK-N-MYC cells were cultured in MEM (#31095 GIBCO) supplemented with 10% FBS, 1% penicillin-streptomycin, 1 mM sodium pyruvate (#ECM0542D EuroClone) and 1% non-essential Amino Acid. For SK-N-MYC cells, 200 µg/ml hygromycinB was added to the medium. IMR-32 cells were cultured in MEM eagle (#M2279 Sigma Aldrich) supplemented with 10% FBS, 1% penicillin-streptomycin, 2 mM L-glutamin and 1% non-essential Amino Acid. HTLA-230 were cultured in DMEM high glucose (#D6546 Sigma Aldrich), supplemented with 10% FBS, 1% penicillin-streptomycin and 2 mM L-glutamin.

For the generation of medulloblastoma neurospheres culture (N-MB), medulloblastoma tissue was explanted from a Math1-cre/Ptch$^{fl/fl}$ mouse and collected in HBBS (#24020083 GIBCO) supplemented with 0.5% glucose and penicillin-streptomycin (#P4333 Sigma Aldrich, St Louis, MO, USA), grossly grinded and treated with DNAse I to a final concentration of 1.28 U/ml for 30'. After centrifugation, single cells were seeded and cultured as neurospheres in a selective medium, as described (Petroni M, Sci Rep 2019). N-MB cells were cultured in DMEM/F12 (#11320033 GIBCO) supplemented with 0.6% glucose (#G8270 Sigma Aldrich), insulin (25 µg/ml, #91077C Sigma Aldrich), N-acetyl-L-cysteine (60 µg/ml, #sc-202232 Santa Cruz), heparin (2 µg/ml, #H3393 Sigma Aldrich), 1% penicillin-streptomycin and B27 supplement without VitA (#12587010 Life Technlogies).

Each cell line was maintained at 37°C in a humidified atmosphere containing 5% CO2.

Each cell line was monitored to exclude mycoplasma infection using PCR Mycoplasma Detection Kit (#G238, Abm, Richmond, BC, Canada).

CRISPR/Cas9

sgRNA specific to the c.8147 T>C mutation (mutATM-sgRNA) and the single-stranded donor oligonucleotides (ssODN) were designed using the informatics platform Benchling (Benchling, 2019, https://benchling.com). PAM sequence (AGG) was located three nucleotides downstream of the c.8147 T>C mutation. ssODN contained the reverted form of the c.8147 T>C mutation and two additional silent "blocking mutations" on the seed sequence to avoid Cas9 re-cutting (Paquet D, *Nature* 2016). The mutATM-sgRNA and scRNA (Origene, Rockville, MD, USA) were cloned into the LentiCRISPR v2 plasmid (Addgene plasmid #52961). IMR-32 cells were co-transfected with mutATM-sgRNA or scRNA-LentiCRISPR v2 plasmid and ssODN using Lipofectamine2000 Transfection Reagent (#11668027 Thermo Fisher Scientific, Waltham, MA, USA) according to the

manufacturer's instructions. To select mutATM-sgRNA/scRNA-LentiCRISPR v2 positive cells, puromycin (1 µg/ml) was added to the medium 24 hours after transfection, for 72 hours. After selection, cells were harvested and seeded in 96-well plates (0.5 cells/well) to obtain monoclonal cell populations. To perform allele-specific PCR screening, DNA was extracted from monoclonal populations using NucleoSpin Tissue (#740952 Macherey-Nagel, Dueren, Germany) according to the manufacturer's instructions. Allele-specific PCR primers were designed on the WT sequence, on the c.8147 T>C mutated sequence and on the sequence of the ssODN (provided in supplemental information). The ssODN positive clones and the scramble clones were sequenced through Sanger method (ABI 3100 Genetic Analyzer, Applied Biosystems, Warrington, UK) to confirm the results. Predicted off-target gene loci were also analyzed by Sanger sequencing to exclude off-target mutations.

In vivo studies

For in vivo experiments, olaparib and MK-8776 were dissolved in DMSO and diluted into 2-hydroxy-propyl-β-cyclodextrin (2-HPβC)/PBS solution just before injection and administered by intraperitoneal (ip) injection at 50 mg/Kg and 25 mg/kg, respectively.

For the subcutaneous xenograft, IMR-32 cells ($4x10^6$ cells) and LAN-5 ($2.5x10^6$ cells) were suspended in an equal volume of medium and matrigel (BD Biosciences, Heidelberg, Germany) and injected in the posterior flanks of 4-weeks-old female BALB/c nude mice (Nu/Nu, Charles River Laboratories, Lecco, Italy). When the tumors reached a size of approximately 150 mm^3, animals were randomly divided into 4 groups and injected with vehicle, olaparib, MK-8776 or their combination and different sets of animals were used to test the early biochemical responses or the long-term efficacy of the drugs. For short term analyses, animals were injected twice with a 24 hours interval (30 hours time point) or every day for 4 days (5 days time point) and then sacrificed to collect tumor tissues for WB or immunohistochemical analysis. Animals for long term experiments were treated every day, 6 days/week. Animals were sacrificed after 14 days of treatment and tumor tissues were collected for weight measurement and macroscopic examination. In survival studies, mice were sacrificed when the tumor size reached 2 cm^3 or at latest on day 40. The time span between the first treatment and the sacrifice was used as a surrogate for survival. MS (50% survival) was determined by GraphPad Prism 7 software (La Jolla, CA). %ILS was calculated by the formula: (median survival of treated mice – median survival of control mice) / median survival of control mice. In all cases, tumor growth was monitored by caliper size measurement and calculated by the formula length x width x 0.5 x (length + width).

For orthotopic xenografts, IMR-32 were transfected with luciferase to obtain IMR-32-luc cells. Five-weeks-old female athymic Foxn1nu mice (Envigo, Bresso, Italy) were subjected to laparotomy and inoculated with $1x10^6$ IMR-32-luc cells into the left adrenal gland capsule, as previously described (Pastorino F, Cancer research 2003). Luciferase activity was visualized by in vivo bio-luminescent imaging (BLI, IVIS Caliper Life Sciences, Hopkinton, MA) after a 10-min incubation with 150 µg/mL of D-luciferin (Caliper Life Sciences), as described (Cossu I, Biomaterials 2015). Tumor-bearing mice were randomized into 4 groups for efficacy studies (n=10 animals/group) and systemic toxicity evaluation (n=3 animals/group). Starting one week after tumor cells implantation, MK-8776 and olaparib, as single agents or in combination, were given every day, 6 days/week, for 4 weeks. Mice were monitored 2-3 times weekly and euthanized before showing signs of illness/suffering, such as paraplegia, dehydration, severe weight loss (>15%) or abdominal dilatation,

according to the ethical committee of the Italian Ministry of Health. The time span between the first treatment and the sacrifice was used as a surrogate for survival.

For the systemic toxicity experiment, mice were anaesthetized with xylezine 48h following the last treatment and blood was collected into either anticoagulant-free tubes or K3EDTA coated tubes through the retro-orbital sinus from each mouse, for clinical chemistry and hematological evaluations. These analyses were performed at the Mouse Clinic, IRCCS Ospedale San Raffaele, Milan. Finally, mice were sacrificed, tumors and healthy organs (liver, spleen, heart, kidney) were recovered, fixed in formalin and embedded in paraffin for subsequent histopathological examination.

For the medulloblastoma mouse model, post-natal day 5 (P5) human GFAP promoter-driven CRE-mediated Ptch-KO (*Ptch*$^{-/-}$) mice (Yang ZJ, Cancer cell 2008) were ip injected with vehicle (n=5), olaparib (n=4), MK-8776 (n=5) or their combination (n=5), every day, for 4 days. Dealing with P5 mice, MK-8776 concentration was further reduced to 12.5 mg/Kg. At the end of the experiment (P9), animals were sacrificed and cerebella were measured by caliper and collected for histopathological examination. Animals were checked regularly, and no signs of illness/suffering were observed until the end of the treatment.

For in vivo experiments Tukey outlier box plot has been used to identify and exclude outliers. Animal experiments were approved by the ethical committee of the Italian Ministry of Health (protocol n.: n°379/2016-PR and n.: 661/2016-PR) in compliance with the "ARRIVE" guidelines (Animals Research: Reporting in Vivo Experiments).

Immunohistochemistry

IHC was performed according to the manufacturer's instructions with the following primary antibodies: a rabbit monoclonal antibody to phosphorylated-H2A.XSer139 #9718 (20E3, Cell Signaling Technology, Massachusetts, USA), a rat monoclonal antibody to CD31 #DIA310 (PECAM-1, clone SZ31, Dianova GmbH, Hamburg, Germany), a rabbit polyclonal antiserum to cleaved caspase-3 #9661 (Asp 175, Cell Signaling Technology, Massachusetts, USA), a mouse monoclonal antibody to KI-67 #M7240 (Clone MIB-1, Agilent, California, USA).

Briefly, antigen-retrieval treatment was applied, when required, using PT link instrument (DakoCytomation, Glostrup, Denmark). Endogenous peroxidase activity was quenched with hydrogen peroxide (3%) for 10 min at room temperature. After blocking with unrelated antiserum, tissue slides were incubated with the primary monoclonal antibodies and/or antiserum in a moist chamber at 4°C. After washing with phosphate buffered saline (PBS), slides analyzed for phosphorylated-H2A.XSer139 and cleaved caspase-3 primary antibodies were incubated with secondary antibodies and EnVision™ FLEX/HRP reagent (Agilent, California, USA). Instead, samples analyzed for anti-CD31 were incubated with a goat anti rat-HRP conjugated antibody (#A24555, TermoFisher Scientific, UK), revealed with 3,3'-diaminobenzidine (Dako EnVision FLEX System; Agilent) as previously reported (Peplau E, Thyroid 2020).

Immunohistochemical staining was assessed and scored by two independent pathologists who were blinded to the clinicopathological data. Discrepancies were resolved by consensus.

List of oligonucleotides and primers

sgRNA and ssODN sequences

mutATM-sgRNA fw	5'-GGAGAGGAGACAGCTTGCTA-3'
mutATM-sgRNA rv	5'-TAGCAAGCTGTCTCCTCTCC-3'
scRNA fw	5'-GCACTACCAGAGCTAACTCA-3'
scRNA rv	5'- TGAGTTAGCTCTGGTAGTGC-3'
ssODN	5'- TTAAAGCAGAATTTCGCTTAGCAGGAGGTGTAAATTTACCA AAAATAATAGATTGTGTAGGTTCCGATGGCAAGGAGAGAAG ACAGTTGGTTAAGGTGAGCCTTCCCTTCTCTGGCTTAGCCCT TAGAGTTTTAGTGATGAAAATTTTTAGTTCATATTTTCTTTCT GCTTTATTTGGGAT -3'

List of PCR allele-specific primers

WT allele fw	5'-GCAAGGAGAGGAGACAGCTT-3'
WT allele rv	5'-AGAGCTCGTCAGCAAATGGT-3'
Mutated allele fw	5'-AAGGAGAGGAGACAGCTTGC-3'
Mutated allele rv	5'-GCACCTCTTGACAGTCTCCC-3'
Reverted allele fw	5'-GCAAGGAGAGAAGACAGTTG-3'
Reverted allele rv	5'-TGTGCACCTCTTGACAGTCT-3'

List of WB antibodies

Primary antibodies used were the following: rabbit anti-phosphorylated CHK1Ser345 #2348 1:1000 in TBS-T with 5% BSA, rabbit anti-phosphorylated P53Ser15 #9284 1:500 in TBS-T with 5% BSA, rabbit anti-ATM (D2E2) #2873 1:500 in TBS-T with 5% BSA, rabbit anti-PARP1 #9542 1:1000 in TBS-T with 5% NFDM, rabbit anti-phosphorylated-H2A.XSer139 #2577 1:500 in TBS-T with 5% BSA, rabbit anti-phosphorylated CHK1Ser296 (D3O9F) #90178 1:500 in TBS-T with 5% BSA (Cell Signaling Technology, Danvers, MA, USA); rabbit anti-phosphorylated-ATMSer1981 (10H11.E12) #ab36810 1:500 in TBS-T with 5% NFDM (Abcam, Cambridge, UK); mouse anti-MYCN #sc-53993 1:500 in TBS-T with 5% NFDM, mouse anti-P53(DOI) #sc-126 1:1000 in TBS-T with 5% NFDM, mouse anti-CHK1 (G-4) #sc-8408 1:1000 in TBS-T with 5% NFDM, goat anti-β-actin (I-19) #sc-1616 1:1000 in TBS-T with 5% NFDM (Santa Cruz Biotechnology, INC, Dallas, TX, USA); rabbit anti-PAR #4336BPC-100 1:500 in TBS-T with 5% NFDM (Trevigen, INC).

Secondary antibodies used were the following: mouse anti-rabbit IgG-HRP #sc-2357 1:5000 in TBS-T with 2.5% NFDM, goat anti-mouse IgG-HRP #sc-2005 1:4000 in TBS-T with 5% NFDM,

donkey anti-goat IgG-HRP #sc-2020 1:5000 in TBS-T with 5% NFDM (Santa Cruz Biotechnology, INC, Dallas, TX, USA).
