# Supplementary material for: A combination of PARP and CHK1 inhibitors efficiently antagonizes MYCN-driven tumors
Source: Oncogene. 2021 Sep 10;40(43):6143–52. doi: 10.1038/s41388-021-02003-0 (PMC8553625; doi:10.1038/s41388-021-02003-0)
Supplement: Supplementary file 2 — Supplementary figures [file 41388_2021_2003_MOESM2_ESM.pdf]

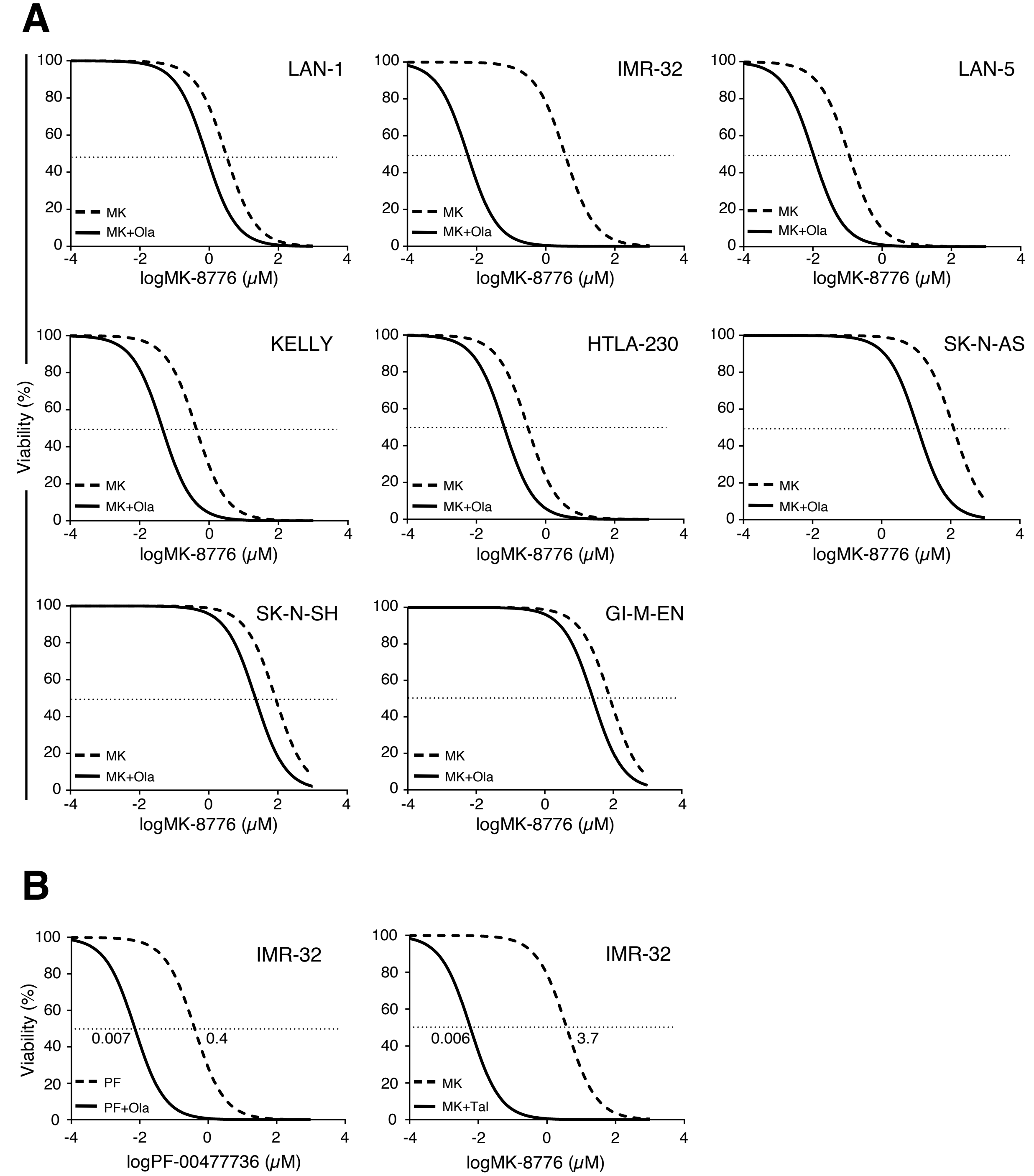

Effects of different combinations of CHK1 and PARP inhibitors on cell viability.

A, Viability assay performed on MNA (LAN-1, IMR-32, LAN-5, Kelly and HTLA-230) and non-MNA (SK-N-SH, SK-N-AS and GI-ME-N) cells after continuous treatment with different doses of MK-8776 alone or in combination with olaparib (10 μM), for 72 hours. Cell viability was calculated as percentage of untreated controls. B, Viability assay performed on IMR-32 after continuous treatment with different doses of the CHK1i PF-00477736 alone or in combination with olaparib (10 μM), and different doses of MK-8776 alone or in combination with the PARPi talazoparib (10 μM), for 72 hours. Cell viability was reported as the percentage of untreated controls. PF-00477736 and MK-8776 IC50 values (μM) are reported close to each curve. Data are expressed as the mean of three independent experiments.

Di Giulio et al.
Supplementary Figure 1

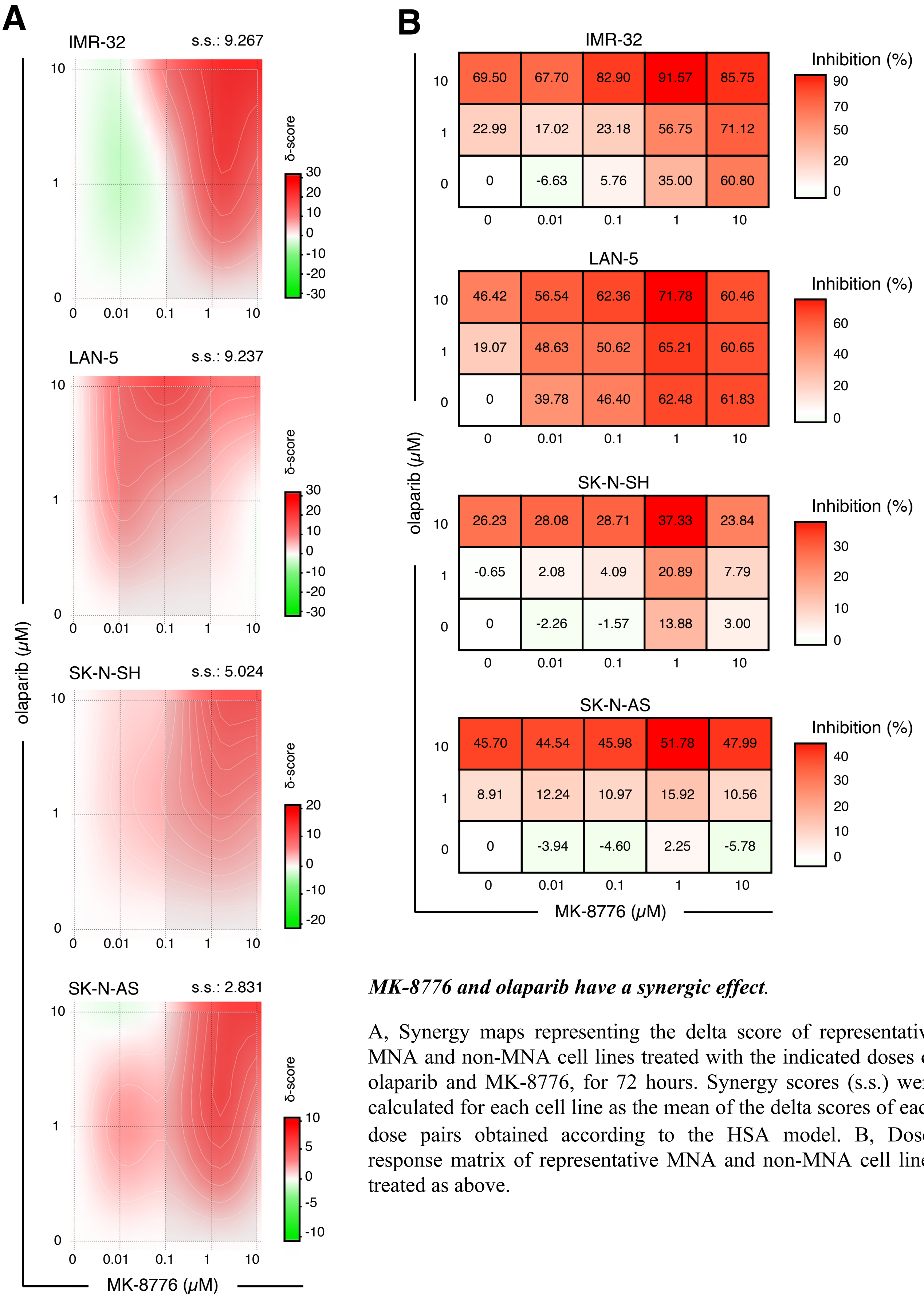

MK-8776 and olaparib have a synergic effect.

A, Synergy maps representing the delta score of representative MNA and non-MNA cell lines treated with the indicated doses of olaparib and MK-8776, for 72 hours. Synergy scores (s.s.) were calculated for each cell line as the mean of the delta scores of each dose pairs obtained according to the HSA model. B, Dose-response matrix of representative MNA and non-MNA cell lines treated as above.

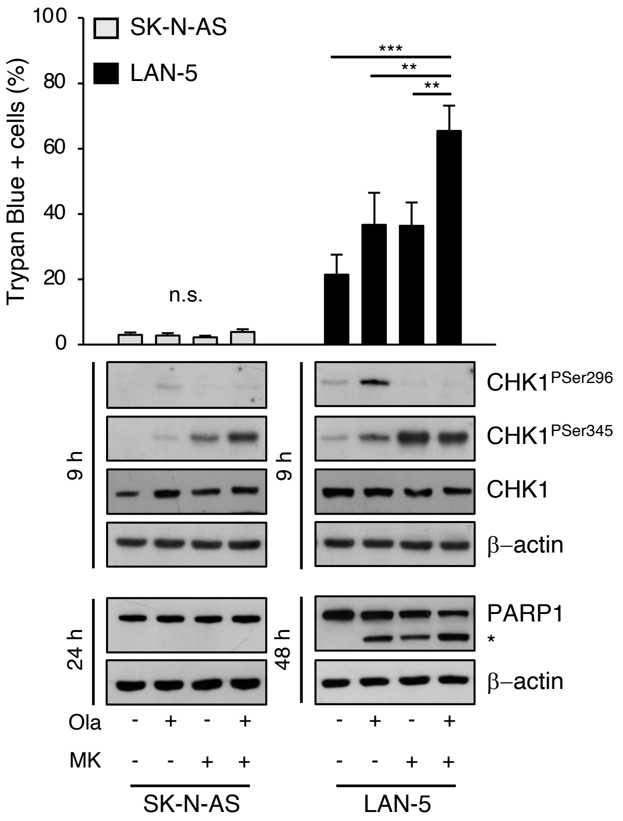

MK-8776/olaparib combination induces cell death in MNA cells.

Evaluation of cell death measured by trypan-blue exclusion assay of SK-N-AS (non-MNA) and LAN-5 (MNA) cells in response to vehicle (DMSO), olaparib (Ola), MK-8776 (MK) and their combination (MK+Ola), at the indicated time points (upper panel; data are reported as mean +SD of three independent experiments). Western blot analysis of the indicated proteins and phosphoepitops (bottom panel) from cells treated as above, for the indicated time points. LAN-5 were treated for longer times compared to other cells because of their longer duplication time. β-actin was used as loading control. The asterisk indicates the 89-kDa PARP1 cleaved fragment. p values were calculated by ANOVA (**$p < 0.01$, ***$p < 0.001$).

Di Giulio et al.
Supplementary Figure 3

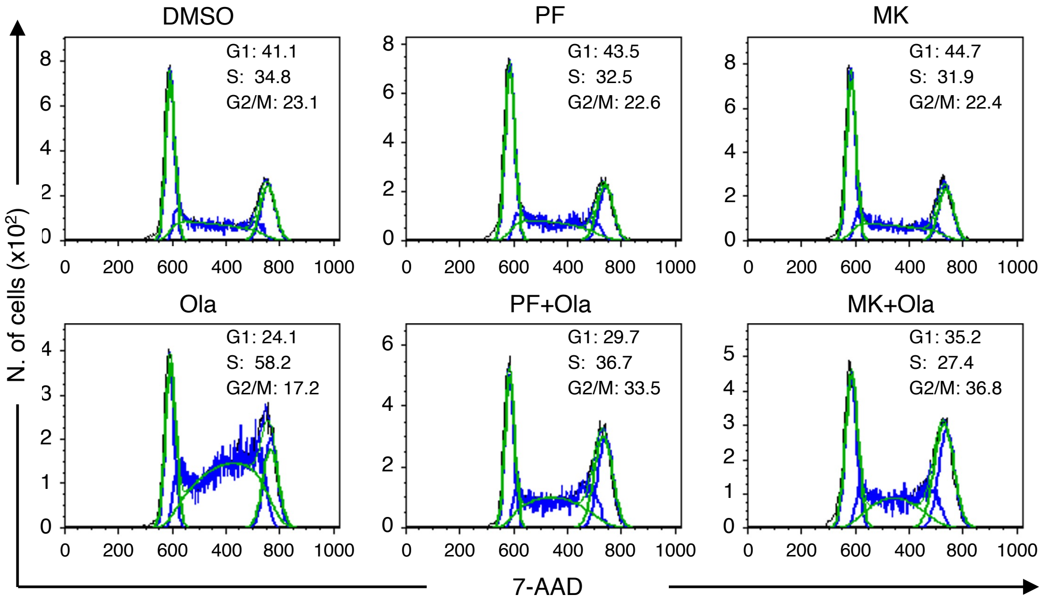

***CHK1 inhibitors abrogate the S-phase checkpoint raised by olaparib*.**

Flow cytometry analysis of DNA content (7-AAD) in MYCN+ cells treated with DMSO, olaparib (Ola), PF-00477736 (PF), MK-8776 (MK) and their combinations (PF+Ola, MK+Ola) for 9 hours. Cell cycle profiles are reported according to Watson Pragmatic (blue line) and Dean Jett Fox (green line) models. The percentage of cells in each phase of the cell cycle was calculated according to Dean Jett Fox model. Data are representative of at least three independent replicates.

Di Giulio et al.
Supplementary Figure 4

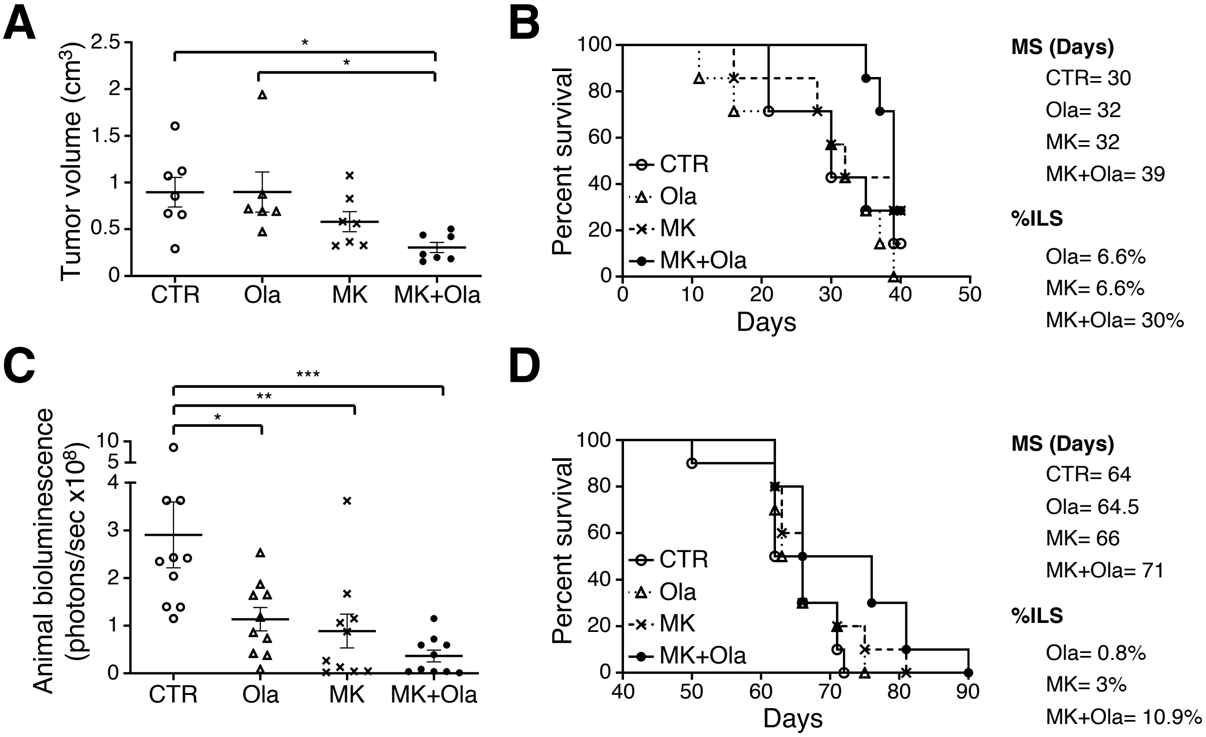

MK-8776/olaparib combination in subcutaneous and orthotopic xenograft mouse models.

A, Evaluation of tumor growth of IMR-32 xenografts intraperitoneally injected with vehicle (CTR), olaparib (Ola), MK-8776 (MK) and their combination (MK+Ola). Data were reported as mean volume (± SEM) after two weeks of treatment (n=7/group, Ola n=6). P values were calculated by ANOVA (*p < 0.05).

B, Survival curve of IMR-32 xenografts. Mice were sacrificed when tumor reached a volume ≥2 cm^3 or alternatively after 40 days of treatment. MS, median survival; %ILS, increase of lifespan compared to controls.

C, Evaluation of tumor growth in nude mice orthotopically inoculated with IMR-32-luc cells and intraperitoneally injected with vehicle (CTR), olaparib (Ola), MK-8776 (MK) and their combination (MK+ Ola). Dot plot representing the photon counts in the tumor region of interest (ROI). Data were reported as mean (± SEM) after three weeks of treatment (n=10/group). P values were calculated by ANOVA (*p < 0.05, **p < 0.01, ***p < 0.001).

D, Survival curve of IMR-32-luc orthotopic xenografts and treated as above. Mice were sacrificed on day 90 or at the earliest sign of illness/suffering. MK-8776/olaparib combination led to a prolonged survival, with a significant increase versus both vehicle (P=0.0192) and single agent olaparib (P=0.0275). MS, median survival; %ILS, increase of lifespan compared to controls.

Di Giulio et al.
Supplementary Figure 5

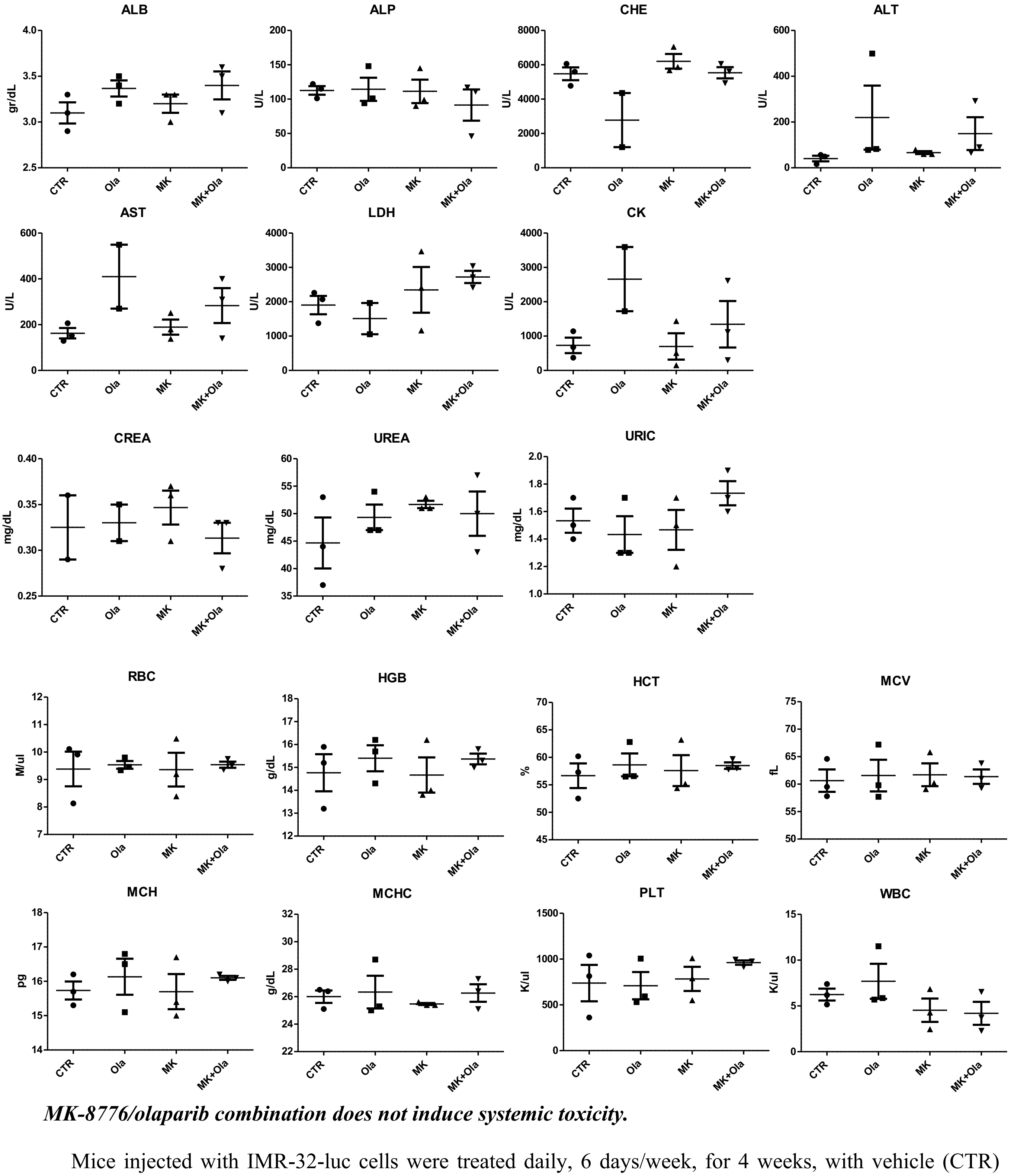

MK-8776/olaparib combination does not induce systemic toxicity.

Mice injected with IMR-32-luc cells were treated daily, 6 days/week, for 4 weeks, with vehicle (CTR) MK-8776 (MK) and olaparib (Ola), as single agents or in combination (MK+Ola) (n=3/group). On appropriately collected blood samples the following parameters were measure: serum albumin (ALB), phosphatase alkaline (ALP), cholinesterase (CHE), glutamic-pyruvic transaminase (ALT), glutamic oxaloacetic transaminase (AST), lactate dehydrogenase (LDH), creatine phosphokinase (CK), creatinine (CREA), UREA nitrogen (UREA) and uric acid (URIC), red blood cells (RBC), hemoglobin (HGB), hematocrit (HCT), mean cell volume (MCV), mean cell hemoglobin (MCH), mean cell hemoglobin concentration (MCHC), platelets (PLT) and white blood cells (WBC). Data are reported as mean (± SEM).

Di Giulio et al.
Supplementary Figure 6

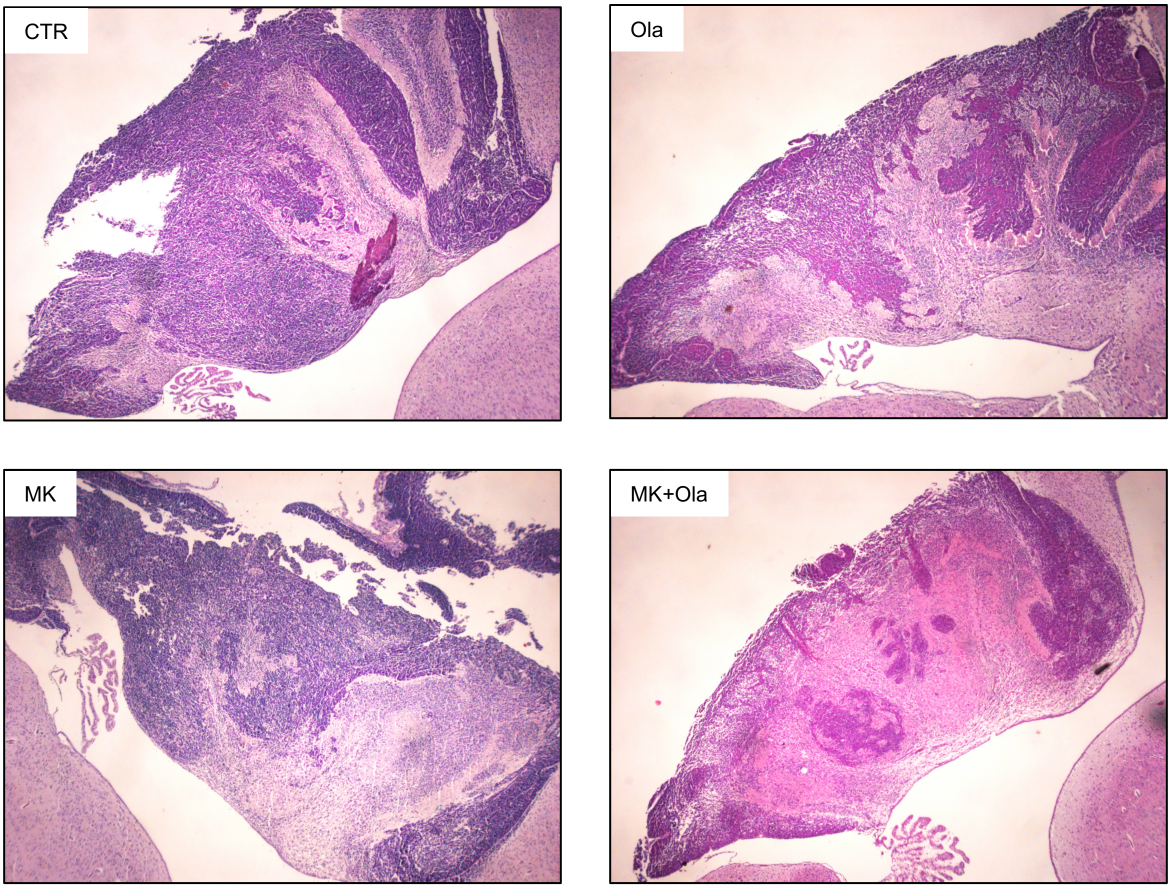

MK-8776/olaparib combination reduces tumor cells in a SHH-dependent medulloblastoma mouse model.

Representative sagittal sections of P9 cerebella from *Ptch*$^{-/-}$ mice after i.p. injection of vehicle (CTR) or olaparib (OLA) or MK-8776 (MK) or their combination (MK+Ola) stained with hematoxylin-eosin (magnification 25x).
